# Contributions of Renin-Angiotensin System-Related Gene Interactions to Obesity in a Chinese Population

**DOI:** 10.1371/journal.pone.0042881

**Published:** 2012-08-06

**Authors:** Jian-Bo Zhou, Chang Liu, Wen-Yan Niu, Zhong Xin, Mei Yu, Jian-Ping Feng, Jin-Kui Yang

**Affiliations:** 1 Department of Endocrinology, Beijing Tongren Hospital, Capital Medical University, Beijing, China; 2 Department of Immunology, Key Laboratory of Immuno Microenvironment and Disease of the Educational Ministry of China, Tianjin Medical University, Tianjin, China; CAEBi, Spain

## Abstract

**Background:**

Gene-gene interactions may be partly responsible for complex traits such as obesity. Increasing evidence suggests that the renin-angiotensin system (RAS) contributes to the etiology of obesity. How the epistasis of genes in the RAS contributes to obesity is still under research. We aim to evaluate the contribution of RAS-related gene interactions to a predisposition of obesity in a Chinese population.

**Methodology and Principal Findings:**

We selected six single nucleotide polymorphisms (SNPs) located in angiotensin (*AGT*), angiotensin converting enzyme (*ACE*), angiotensin type 1 receptor (*AGTR1*), *MAS1*, nitric oxide synthase 3 (*NOS3*) and the bradykinin B2 receptor gene (*BDKRB2*), and genotyped them in 324 unrelated individuals with obesity (BMI ≥28 kg/m^2^) and 373 non-obese controls (BMI 18.5 to <24 kg/m^2^) from a large scale population-based cohort. We analyzed gene-gene interactions among 6 polymorphic loci using the Generalized Multifactor Dimensionality Reduction (GMDR) method, which has been shown to be effective for detecting gene-gene interactions in case-control studies with relatively small samples. Then we used logistic regression models to confirm the best combination of loci identified in the GMDR. It showed a significant gene-gene interaction between the *rs220721* polymorphism in the *MAS1* gene and the *rs1799722* polymorphism in the gene *BDKB2R*. The best two-locus combination scored 9 for cross-validation consistency and 9 for sign test (p = 0.0107). This interaction showed the maximum consistency and minimum prediction error among all gene-gene interaction models evaluated. Moreover, the combination of the *MAS1 rs220721* and the *BDKRB2 rs1799722* was associated with a significantly increased risk of obesity (OR 1.82, CI 95%: 1.15–2.88, p = 0.0103).

**Conclusions and Significance:**

These results suggest that the SNPs from the RAS-related genes may contribute to the risk of obesity in an interactive manner in a Chinese population. The gene-gene interaction may serve as a novel area for obesity research.

## Introduction

It has been argued that gene-gene interaction is a ubiquitous component of the genetic architecture of common human diseases, such as obesity [1]. Although genome-wide association studies have shown robust associations between susceptibility loci and obesity [2], [3], [4], [5], [6], [7], these loci explain no more than 2% of the individual variation in the susceptibility of obesity [6]. The complexity of obesity may be explained in part by complex gene-gene interaction or epistasis [8], [9], [10]. Moreover, interaction between genes plays an important role in determining the severity of obesity [11]. Family studies estimate the genetic heritability of obesity at ∼40%, and twin studies place the figure higher, at ∼65% [12]. The consistent differences in these estimates suggest that as much as one third of the heritable variance may be due to non-additive genetic variance, including allelic (dominance and recessivity) and non-allelic gene interactions. Therefore, describing gene-gene interactions is imperative to characterizing certain obesity-related traits, particularly when each involved locus only demonstrates a minor marginal effect.

Increasing evidence suggests that the renin-angiotensin system (RAS) contributes to the etiology of obesity. The renin-angiotensin system is involved in adipocyte growth and differentiation and possibly in adipose tissue metabolism. Hyperactivity of systemic and tissue-specific RAS is related to increased propensity to obesity [13], [14]. Genetic intervention of RAS components inhibits the development of obesity in rodents [15], [16], [17]. Genetic epidemic investigations of associations between RAS polymorphisms and obesity have generated mixed results in human studies [18], [19]. An association of body mass index (BMI) with angiotensin converting enzyme insertion/deletion (ACE I/D) (P = 0.035), whereas no association with angiotensinogen M235T and angiotensin II receptor 1 (AGTR1) A1166C gene polymorphisms was obtained in a Tunisian population [20]. While, angiotensin converting enzyme insertion/deletion, angiotensinogen M235T and angiotensin II receptor 1 (AGTR1) A1166C gene polymorphisms were associated with the body mass index in a Romania population [19]. The underlying genetic contribution to obesity from the gene-gene interaction of RAS still remains unclear. The contribution of epistasis from RAS-related genes to complex traits such as hypertension and atherosclerosis were explored in recent studies [21], [22]. The aim of our study was to explore the contribution of epistasis among RAS related genes, including AGT, ACE, ATR1, MAS1, NOS3 and bradykinin receptor 2 (BDKRB2) genes. In this study, we genotyped 6 different loci in the 6 RAS-related genes in unrelated obesity and non-obesity control subjects, and examined gene to gene interactions with the generalized multifactor dimensionality reduction (GMDR) method.

## Materials and Methods

### Ethics statement

The study was conducted with the approval from the Ethics Committee of Beijing Tongren Hospital, Capital Medical University. Written informed consent was obtained from each participant.

### Study population

Study subjects were selected from a completed large scale population-based cohort (the Beijing Community Pre-Diabetes Study) designed to facilitate the conduct of genetic epidemiology investigations and clinical trials on individuals originating from the settled community of Nanfaxin, a satellite rural town of Beijing. Demographic data were depicted in details in our published paper [23]. All the individuals were given a normal diet for three days before the survey. All subjects were invited to attend a baseline health examination. This included anthropometric data, including body mass index (BMI), waist circumference and blood pressure. In addition, a general health questionnaire, with questions on personal and family history of disease and lifestyle factors was completed. According to the diagnosis of obesity from the Working Group on Obesity in China (WGOC, 6), 324 subjects with BMI ≥28 kg/m^2^ were defined as obese, while 373 subjects with BMI from 18.5 to <24 kg/m^2^ were chosen as the group of control individuals. Both groups had comparable blood pressure, serum creatinine, fasting plasma glucose (FPG) and age. Subjects with secondary obesity, coronary heart disease and chronic kidney disease were excluded from the study.

### Laboratory measurements

Biochemical parameters were measured by Beckman-Unicel Dxc800 biochemistry analysator (Beckman Ltd, CA, USA) including FPG, total cholesterol (TC), triglycerides (TG), low-density lipoprotein (LDL), high-density lipoprotein (HDL) cholesterol, alanine aminotransferase (ALT) and creatinine (Cr).

### Gene and SNP selection

Genomic DNA was extracted from peripheral blood leukocytes using standard methods. This set of SNPs was chosen based on the following criteria: HapMap validation status, functional relevance and importance [11], [18], [19], [24], [25], [26], and a minor allele frequency (MAF) exceeding 5% (HapMap CHB databank).. SNPs from six RAS-related genes were assessed: rs699 of the angiotensin (AGT) gene, insertion/deletion (I/D) polymorphism of the angiotensin converting enzyme (ACE) gene, rs5186 of the angiotensin type 1 receptor (AGTR1) gene, rs220721 of the MAS1 gene, rs1799983 of the nitric oxide synthase 3 (NOS3) gene, and rs1799722 of the bradykinin receptor 2 (BDKRB2) gene.

### Genotyping of polymorphisms

The genotyping was performed using primer extension of six loci. Detection was completed by a matrix-assisted laser desorption/ionisation time-of-flight mass spectroscopy using a MassARRAY platform (MassARRAY Compact Analyzer, Sequenom, San Diego, CA, USA). 5% of the samples were duplicated to evaluate the genotyping concordance rate. Genotypes between duplicates were concordant for all six SNPs, and no evidence of departure from Hardy-Weinberg equilibrium was observed in control subjects using chi-squared-test analysis.

### Statistical analysis

Statistical analysis was performed using SPSS version 13.0 for Windows (SPSS Inc., Chicago, USA). Data are presented as numbers or means ± SD as appropriate. The between-group data were compared with Student's unpaired t-test for continuous data and with the chi-squared-test for categorical data. Allele frequencies were calculated from the genotypes of the subjects. Differences in allele and genotype frequencies between cases and controls were compared with a chi-squared-test or a Fisher's exact test.

To identify higher order gene-gene interactions in our samples, we used the MDR software 2.0-beta (http://www.multifactordimensionalityreduction.org). All possible two-, three-, four- and five-way SNP interactions were tested using 10-fold cross-validation in an exhaustive search. The MDR method is described in detail elsewhere [8]. Briefly, in an analysis of n-way interactions, the n-dimensional space formed by all possible combinations of values (classes) of a given set of n variables (in this case, SNPs) is reduced to a single dimension by reclassifying each class as either high risk or low risk according to the relative proportion of cases to controls in that class. This statistical method included a 10-fold cross-validation and permutation-testing procedure to minimize false positive results by multiple examinations of the data. With 10-fold cross-validation, the data are divided into 10 equal parts, and the model is developed on 9/10 of the data (training set) and then tested on 1/10 of the remaining data (testing set). This is repeated for each possible 9/10 and 1/10 of the data, and the resulting 10 prediction errors are averaged. The combination with the lowest prediction error is reported. Finally, hypothesis testing for this selected model can then be performed by evaluating the consistency of the model across cross-validation data sets. Under the null hypothesis that no association was derived from 1000 permutations, the average cross-validation consistency from the observed data was compared to the distribution of average consistencies. The null hypothesis was rejected when the P-value derived from the permutations was ≤0.05.Generalized multifactor dimensionality reduction (GMDR) is based on the score of a generalized linear model, of which the original MDR method is a special case [27]. Investigating multiple SNPs necessitates careful consideration of multiple comparison issues, which is exactly the benefit of using the GMDR approach. We then employed logistic regression models to confirm the best combination of loci identified in the GMDR. Power analysis for case-control samples was carried out by Armitage's test for trend. (alpha has been set at 0.05) [28].

## Results

The characteristics of the study subjects are provided in Table 1. The distributions of age and sex between case and control individuals were well matched. Similar distributions were observed for systolic blood pressure, diastolic blood pressure, FPG and serum creatinine between the two groups. As expected, significance was noted for total cholesterol, high-density lipoprotein, low-density lipoprotein and triglyceride (p<0.01). There was no deviation from the Hardy-Weinberg Equilibrium for all studied polymorphisms in controls (p>0.05).

**Table 1:** Clinical characteristics of the participants.

Characteristic	Obesity	Control	P-value
n	324	373	
Men/Women	138/186	166/207	0.612
Age (years)	55.73±9.96	56.59±11.49	0.294
BMI (kg/m^2^)	30.65±2.40	21.82±1.64	<0.001
SBP (mmHg)	132.59±18.54	130.11±20.00	0.433
DBP (mmHg)	80.26±10.14	78.0±10.46	0.299
TC (mmol/l)	4.79±0.94	4.48±0.96	<0.01
TG (mmol/l)	1.78(16.45)	0.99(4.79)	<0.001
HDL-c (mmol/l)	1.10±0.26	1.26±0.29	<0.001
LDL-c (mmol/l)	3.07±0.95	2.79±0.82	<0.001
Cr (µmol/l)	61.05±20.98	60.39±19.57	0.384
FPG (mmol/l)	6.96±2.41	6.47±2.36	0.356
Smoking (%)			
Never	83.1	76.1	
Former or current	16.9	23.9	0.029
Drinking (%)	12.2	14.3	0.421

Age, body mass index (BMI), systolic blood pressure (SBP), diastolic blood pressure (DBP), TC, HDL-c, LDL-c, Cr and fast plasma glucose (FPG) values are given as mean (SD); TG values are given as median (range).

The GMDR was used to assess the contribution of combinations of the six variants to the etiology of obesity including the age and sex as the covariates. Table 2 summarizes the results from the GMDR analysis for one locus to six-loci with obesity. With co-variable adjustments, the best combination was the two-locus model, *MAS1 rs220721* and *BDKB2R rs1799722*, suggesting the two variants together contributed to the etiology of obesity. The two-locus model had the highest level of testing balance accuracy (0.5601), the maximum cross-validation consistency, and scored 9 for the sign test at the 0.0107 level. The two-locus genotype combinations were classified into high- or low-risk groups. In the chi-squared test, the OR of high-risk combination of the two-locus model increased the risk of obesity by 1.82 times (95% CI: 1.15 to 2.88, p = 0.0103).

**Table 2:** GMDR results of multi-locus interaction with obesity and BMI.

No. of loci with Obesity	Best Model *	Training Balance Accuracy	Testing Balance Accuracy	Cross-validation consistency	Sign Test (P-value)
1	BDKRB2	0.5459	0.5194	6	7(0.1719)
2	**MAS1, BDKRB2**	**0.5653**	**0.5601**	**10**	**9(0.0107)**
3	ACE, MAS1, NOS	0.5838	0.5022	4	5(0.6230)
4	ACE, MAS1, BDKRB2, NOS	0.6081	0.4848	7	6(0.3770)
5	MAS1, AGT, ACE, BDKRB2, NOS	0.6429	0.5019	10	6(0.3770)
6	MAS1, AGT, ACE, AGTR1, BDKRB2, NOS	0.6634	0.4855	10	4(0.8281)

No. of loci with BMI	Best Model *	Training Balance Accuracy	Testing Balance Accuracy	Cross-validation consistency	Sign Test(p)
1	MAS1	0.5486	0.5271	8	7(0.1719)
2	**MAS1, BDKRB2**	**0.5749**	**0.5681**	**10**	**10(0.0010)**
3	MAS1, AGT, NOS	0.5922	0.5566	8	8(0.0547)
4	MAS1, ACE, BDKRB2, NOS	0.6148	0.5189	7	6(0.3770)
5	MAS1, AGT, ACE, BDKRB2, NOS	0.6489	0.5261	10	6(0.3770)
6	MAS1, AGT, ACE, AGTR1, BDKRB2, NOS	0.6691	0.5308	10	7(0.1719)

* Adjusted by age and sex.

In addition, BMI as the continuous variable was analyzed to explore the relationship with the combination of the six variants (Table 2). With co-variable adjustments, there was a significant two-locus model involving *MAS1 rs220721* and *BDKRB2 rs1799722*, as well as a marginal significant three-locus model involving *MAS1 rs220721*, *AGT rs699* and *NOS3 rs1799983*. Overall, the two-locus model had 10 cross-validation consistency, the highest testing balance accuracy of 0.5681 and 10 for the sign test (p = 0.001), which was the same best model as that which was identified with BMI as two-classified variable. The two-locus model was thus chosen as the best model.

Since the GMDR was based on the MDR, analysis from the MDR was also performed. The accuracy of the two-locus model using the same SNPs without co-variable adjustment was similar to that with adjustment (see Table 3).

**Table 3:** Comparison of best models of MDR and GMDR for predicting obesity.

	Training Balance Accuracy	Testing Balance Accuracy	Cross-validation consistency	Sign Test (P-value)
MDR	0.5658	0.5620	10	0.0010
GMDR	0.5653	0.5601	9	0.0107

To further classify the interactions of genetic variants on the disposition of obesity, the following analysis was explored on the basis of two variants identified by GMDR. Results from the logistic regression analysis showed that *MAS1 rs220721* variant was significantly and positively associated with the predisposition of obesity under both the allelic genetic model (OR = 1.31, 95% CI: 1.04, 1.64, p = 0.023), and the recessive genetic model (OR = 1.40, 95% CI: 1.03, 1.89, p = 0.032) even after adjusting for other confounders (age and sex or plus smoking and FPG) (Table 4). Under both dominant and allelic models of inheritance, a similar tendency was observed for the *BDKRB2 rs1799722* polymorphism (OR = 1.75, 95% CI: 1.18, 2.58, p = 0.005 for the dominant model of inheritance; OR = 1.58, 95% CI: 1.09, 2.29 p = 0.015 for the allelic model of inheritance). Comparison of the genotype combinations of *MAS1 rs220721 GG* and *BDKRB2 rs1799722 CC*, an increased number of risk alleles correlated with a significantly increased risk of obesity (OR 1.43, 95% CI: 1.17, 1.77, p = 0.001). There was a non-significant gene-gene interaction of deviation in a multiplicative manner in the logistic regression model.

**Table 4:** The association between three genetic models for six variants of six genes and obesity.

	Dominant model		Recessive model		Allelic model	
	OR#	OR*	OR#	OR*	OR#	OR*
**AGT:rs669**	1.16 [0.85–1.57] (0.352)	1.11 [0.81–1.54] (0.509)	0.89 [0.44–1.78] (0.742)	0.77 [0.35–1.68] (0.512)	1.09 [0.84–1.40] (0.513)	1.05 [0.79–1.37] (0.746)
**ACE:I/D**	1.11 [0.82–1.51] (0.490)	1.20 [0.87–1.66] (0.262)	0.94 [0.61–1.46] (0.792)	1.08 [0.69–1.71] (0.730)	1.04 [0.84–1.29] (0.718)	1.12 [0.89–1.41] (0.330)
**ATR1:rs5186**	–	–	1.11 [0.69–1.76] (0.669)	1.27 [0.77–2.09] (0.343)	1.11 [0.69–1.76] (0.669)	1.27 [0.77–2.09] (0.343)
**MAS1:rs220721**	1.41 [0.87–2.30] (0.163)	1.45 [0.87–2.43] (0.152)	1.40 [1.03–1.89] (0.032)	1.51 [1.09–2.08] (0.013)	1.31 [1.04–1.64] (0.023)	1.37 [1.08–1.75] (0.011)
**BDKRB2: rs1799722**	1.75 [1.18–2.58] (0.005)	1.83 [1.20–2.78] (0.005)	0.58 [1.14–2.36] (0.452)	0.74 [0.18–2.99] (0.670)	1.58 [1.09–2.29] (0.015)	1.66 [1.12–2.46] (0.011)
**NOS3:rs1799983**	1.02 [0.70–1.49] (0.913)	0.98 [0.66–1.46] (0.924)	1.76 [0.29–10.63] (0.540)	1.42 [0.22–8.97] (0.708)	1.04 [0.73–1.49] (0.820)	0.99 [0.69–1.45] (0.990)

Data are expressed as OR [95% CI] (P value).

# Adjusted by age and sex.

* Adjusted by age, sex, FPG and smoking.

## Discussion

Obesity is a complex disease, which is influenced by genetic and environmental factors. Family and twin studies have estimated that 40–70% of the variance in obesity-related traits is attributed to genetic factors [29], [30]. In this study, we investigated the contribution of SNPs from six RAS-related genes to susceptibility. Two genetic variants in MAS1 and BDKRB2 were identified to be significantly associated with obesity risk. The interactions between MAS1 and BDKRB2 were identified based on GMDR analysis. Our findings, for the first time, suggested that the interaction between *MAS1* and *BDKRB2* conferred the genetic susceptibility to obesity in the Chinese population. We consider the present study to be explorative and hypothesis-generating and thus find the results intriguing. However, a more convincing conclusion can only be reached by further independent replication of the results reported in this investigation.

In recent years, more and more studies have focused on gene-gene interactions [31], [32], [33], [34], [35], which are partly due to the appearance of new statistical theory. MDR is proved to be a useful statistical tool to detect gene-gene interactions while avoiding “the dimension curse” [36]. However the existing approaches do not allow for covariates. The GMDR, based on the MDR, permits the adjustment of discrete and quantitative covariates, and is applicable to both dichotomous and continuous data. In our study, GMDR suggested that the combination between *MAS1 rs220721* and *BDKRB2 rs1799722* was the best model, irrespective of whether a dichotomous (obesity) or continuous (BMI) variable was measured, adjusting for the covariates. This *SNPs* combination increased the risk of obesity by 1.82 times, which inferred that the gene-gene interaction play a role in the vulnerability of obesity in our study population. In this research, differences were observed in the distribution of polymorphic variants of MAS1 and BDKRB2 between obesity patients and controls. The results of the logistic regression further show that an increased number of risk alleles correlates with a significantly increased risk of obesity, in comparison with the genotype combinations of *MAS1 rs220721 GG* and *BDKRB2 rs1799722 CC*. However, the susceptibility interaction was not confirmed by the logistic regression analysis. The possible reason for these inconsistent results is that GMDR did not detect the interaction defined by “deviation from the multiplicative” in the logistic regression model. The significant results from GMDR only showed that the combination of different loci may increase or decrease the risk of obesity and the interaction among the loci may refer to either multiplicative, deviation from the multiplicative or departure from additivity [37], [38].

Activation of the RAS in adipose tissue has been implicated in the regulation of adiposity and overt obesity through its ability to increase fat cell growth and differentiation, increase synthesis, uptake and storage of fatty acids and triglycerides [39]. Many researches also were focused on the association of single polymorphism from RAS with the obesity [11], [18], [19], [20], [24], [25]. The results were conflicting. The contributions of gene-gene interaction to the disposition of obesity maybe supply an explanation. It is also worth mentioning that gene-gene interaction has been reported at the cellular level [40], which suggests that deep insight from the statistical level is relevant at the biological level. Additionally, this is the first attempt at a systems biology approach to integrate clinical observations with SNPs in obesity-related genes. Our study had some limitations. Firstly, our study involved a number of comparisons, and associations arising out of chance must be considered as a possible explanation for statistically significant results. A further limitation is that a true disease-associated gene may have multiple functional variants. By representing each gene locus with a single SNP, we are therefore missing out on the potential effects of multiple association signals for a given gene. A better gene statistic would evaluate all the variation across a locus, rather than just the SNP with the best association statistic, while accounting for any LD [41]. Thirdly, it may be difficult to interpret GMDR results. Finding out the essence of epistasis in multidimensional space to infer function remains an interpretive challenge. Maybe it is practicable to seek the biological explanations for such an epistasis by considering various physiological effects of the researched polymorphisms [27]. Although the discovery of epistasis in our research may be of limited value for elucidating the underlying biological disease process, different modes of interaction between potential disease loci can lead to improved power for detection of genetic effects.

In conclusion, our study is the first to show evidence that the interaction between *MAS1* and *BDKRB2* is associated with the predisposition of obesity in our Chinese population. We believe these findings will guide further investigations in gene detection and may shed light on the genetic architecture of obesity.
